# Pharmacological Studies on Neuromodulatory Effects of Plant Extracts

**DOI:** 10.3390/ijms241310653

**Published:** 2023-06-26

**Authors:** Luigi Brunetti

**Affiliations:** Department of Pharmacy, G. d’Annunzio University of Chieti-Pescara, 66013 Chieti, Italy; luigi.brunetti@unich.it

Central nervous system (CNS) disorders represent a public health priority and demand significant scientific efforts for the development and study of new drugs and their possible beneficial effects. It has been estimated that CNS disorders result in death in one of every nine patients; these disorders include ictus, Parkinson’s disease (PD), and Alzheimer’s (AD) disease [1]. There is a large amount of interest in natural compounds as, particularly in CNS disorders. In particular, various studies suggested that natural products can improve mood states, neuroinflammation, and neurodegenerative diseases [2,3]. This Special Issue contains six papers and several reviews which explore the neuroprotective effects of potential new drugs from plant sources. The contributions covered a variety of CNS disorders, exploiting different experimental approaches.

Yokubaitis and collaborators [4] studied the protective effects of beta-caryophyllene (BCP), a sesquiterpene found in *Cannabis sativa*, black pepper, clove, and rosemary, and of phytocannabinoid cannabidiol (CBP) on a model of permanent cerebral ischemia. In particular, the authors focused their attention on the potential interactive effects between BCP and CBP. The potential neuroprotective effects induced by the combination of CBD and BCP was attributed to their selective binding to cannabinoid receptor (CB)1 and CB2, respectively [5,6,7,8]. This would entail an improvement in therapeutic index and allow the use of lower doses of one or both of the combination constituents. Consistently, a synergistic analgesic effect of a CBD and CBP combination has also been reported by Blanton and collaborators [9]. Although further studies are needed to determine the real benefits of this association, the same authors provided potential mechanisms. The capacity of cannabinoids and terpenes to work synergistically is fascinating and this combination could offer a valid therapeutic approach in neurodegenerative diseases. These results set the stage for future studies that could evaluate the potential effects exerted by the combination of other terpenes and cannabinoids, including cannabigerol (CBG). CBG, a non-psychoactive phytocannabinoid, showed beneficial effects on different CNS disorders [6,7,8]. In addition, preclinical studies demonstrated that CBG represents a valid neuroprotective agent [10,11,12].

In this context, various comparative in vitro studies of CBD and CBG were reported [13,14]. However, di Giacomo and collaborators reported that CBG was effective at lower concentrations compared to CBD, particularly for astrocytes and isolated prefrontal cortices from rats exposed to hydrogen peroxide. Using bioinformatic and docking analyses, the same authors hypothesized for the first time a possible involvement of neurokinin B receptor (NK3R) in the neuroprotection induced by CBG [14]. Based on these results, CBG could be a treatment for mood disorders since NK3R represents a possible target in psychiatric diseases, including schizophrenia and affective disorders [15].

Typically, treatment of emotional disorders includes a combination of drugs, as well as psychotherapy and family support. Dietary supplements, including herbal or plant-based extracts, micronutrients, and food-based nutraceuticals are widely used in different neurological diseases, such as mood and cognitive function disorders [16]. An important opportunity in this field is the possibility of combining natural products, as demonstrated by Ano and collaborators. Recently, these authors showed that supplementation with hop bitter acids such as iso-α-acids (IAA) and mature hop bitter acids (MHBA) improved mood in adults [17]. This effect could be related to increased dopaminergic activity in cerebral areas, such as the prefrontal cortex and hippocampus [17]. Furthermore, Kung and Li [18] reviewed the therapeutic role of alpha-eleostearic acid (α-ESA), an isomer of conjugated linolenic acids derived from wild bitter melon (*Momordica charantia* L. var. abbreviata Ser.), and curcumin in CNS diseases. The authors also discussed the potential involvement of glia-mediated neuroinflammation and mitochondrial dysfunction along with CDGSH iron-sulfur domain 2 (CISD2) decline and NFκB activation in CNS injuries and diseases. Their review improves our understanding of the CNS pathology–CISD2–NFκB axis and clarify perspectives on the potential role of these natural compounds as therapeutic agents [18].

In another review, Kim explored the neuroprotective effects of sulforaphane (SFN) on cognitive disorders, including AD, Parkinson’s disease, Huntington’s disease, amyotrophic lateral sclerosis, multiple sclerosis, autism spectrum disorder, and schizophrenia. Kim discussed the anti-AD-like activity of sulforaphane and how it decreased levels of AD biomarkers, such as amyloid-β, tau, inflammation, oxidative stress, and neurodegeneration in AD-like animal and cell models. In addition, the author focused on potential mechanisms involved in the neuroprotective effects induced by SFN. This review demonstrates that SFN has a multifaceted neuroprotective effect on AD pathophysiology, indicating the need to pursue SFN research [19].

Among natural products, triterpenoid acids also play a crucial role in CNS disorders, through their capacity to modulate several signaling pathways involved in neuro-inflammation and apoptotic processes. In their review, Gudoityte and collaborators focus on ursolic (UA) and oleanolic acids (OA). The neuroprotective activity of UA/OA and their derivatives could be related to stimulation of cellular antioxidant defenses [20]. In summary, all the research articles and reviews in this Special Issue provide new insights to allow us to further investigate the role of novel natural compounds in CNS diseases.
